# Ovarian Tuberculosis Mimicking Ovarian Malignancy: A Diagnostic Challenge in an Endemic Setting

**DOI:** 10.7759/cureus.104944

**Published:** 2026-03-09

**Authors:** María E Galaviz Valdez, Paula M Cuéllar Pinzón, Deborah L Núñez

**Affiliations:** 1 Internal Medicine, Hospital Universitario Dr. José Eleuterio González, Monterrey, MEX

**Keywords:** adnexal mass, ca-125, extrapulmonary tuberculosis, female genital tuberculosis, ovarian tuberculosis

## Abstract

Ovarian tuberculosis is a rare manifestation of extrapulmonary tuberculosis and may closely resemble advanced ovarian carcinoma due to overlapping clinical, biochemical, and radiological features. We report the case of an 18-year-old female who presented with progressive abdominal distension, severe hypogastric pain, and unintentional weight loss. Imaging revealed a left adnexal mass with ascites and retroperitoneal lymphadenopathy suggestive of stage IIIC ovarian carcinoma. Serum CA-125 was markedly elevated (637.4 U/mL). Prior to initiation of chemotherapy, an image-guided TRUCUT biopsy demonstrated chronic granulomatous inflammation with acid-fast bacilli. Ziehl-Neelsen staining was positive, QuantiFERON-TB Gold assay was positive, and culture of the adnexal lesion confirmed *Mycobacterium tuberculosis*. The patient was treated with first-line anti-tuberculous therapy and showed clinical and radiological improvement. This case highlights the importance of considering tuberculosis in the differential diagnosis of adnexal masses with elevated CA-125, particularly in endemic regions, and emphasizes the value of histopathological confirmation to prevent misdiagnosis and avoid unnecessary oncologic treatment.

## Introduction

Female genital tuberculosis (FGTB) is an uncommon form of extrapulmonary tuberculosis that represents an important cause of gynecologic morbidity and infertility [1], particularly in countries with a high or intermediate tuberculosis burden, such as Mexico [2]. Extrapulmonary tuberculosis accounts for approximately 15-20% of global tuberculosis cases, with urogenital involvement representing a significant yet frequently underrecognized subset [3].

FGTB typically results from hematogenous dissemination of *Mycobacterium tuberculosis* (MTB) from a primary pulmonary focus. The fallopian tubes are involved in 90-100% of cases, followed by the endometrium (50-80%) and the ovaries (20-30%) [4]. Isolated ovarian involvement is uncommon and may lead to delayed diagnosis or misinterpretation as adnexal malignancy [5].

The clinical presentation is often insidious and nonspecific. Many patients are diagnosed during infertility evaluation; however, symptoms may include chronic pelvic pain, menstrual irregularities, abnormal vaginal discharge, or the presence of a pelvic mass [4]. Several case reports have described ovarian tuberculosis presenting as a suspected ovarian malignancy, because of elevated CA-125 levels and complex adnexal lesions on imaging, posing a significant diagnostic challenge [5].

Diagnosis remains difficult due to the paucibacillary nature of the disease, meaning that only a low number of bacteria are present in the sample. Acid-fast bacilli smear has low sensitivity, and mycobacterial culture, while considered the reference standard, may require several weeks for results. Molecular tests such as Xpert MTB/rifampicin (RIF) demonstrate high specificity but variable sensitivity in gynecologic specimens. Consequently, diagnosis often relies on a composite approach integrating clinical findings, imaging studies, histopathology, and microbiological testing [1].

We present the case of a patient with histologically confirmed ovarian tuberculosis whose clinical and radiologic presentation simulated adnexal malignancy, highlighting the diagnostic challenges and the importance of considering tuberculosis in the differential diagnosis of complex ovarian masses in endemic settings.

## Case presentation

An 18-year-old previously healthy female presented in April 2023 with progressive abdominal distension and severe spasmodic hypogastric pain rated 10/10 in intensity. She reported regular menstrual cycles since menarche, with no history of dysmenorrhea, abnormal uterine bleeding, pelvic inflammatory disease, or prior gynecologic conditions. She reported unintentional weight loss of approximately 4 kg over two months. She denied fever, respiratory symptoms, or known tuberculosis exposure.

Abdominal ultrasound revealed a left adnexal mass with free intraperitoneal fluid. Subsequent laboratory evaluation demonstrated a markedly elevated serum CA-125 level of 637.4 U/mL.

**Figure 1:**
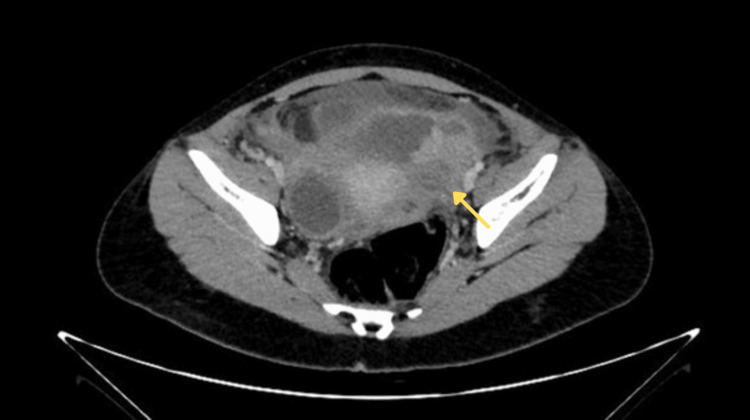
Contrast-enhanced axial computed tomography (CT) scan of the abdomen and pelvis demonstrating a heterogeneous left adnexal mass (arrow) measuring approximately 46 × 38 × 30 mm, associated with peritoneal thickening and ascites.

Contrast-enhanced computed tomography (CT) demonstrated a heterogeneous, partially defined left adnexal mass measuring 46 × 38 × 30 mm, associated with retroperitoneal lymphadenopathy and radiological findings suggestive of peritoneal carcinomatosis. In the right adnexal region, a rounded lesion is identified, with well-defined margins and a thin wall, hypodense, measuring 40 × 31 × 43 mm, with an approximate volume of 28 cc, consistent with an adnexal cyst (Figure 1). Based on these findings, advanced ovarian carcinoma (minimum stage IIIC) was suspected.

Prior to initiating chemotherapy, an image-guided TruCut biopsy was performed. Histopathological examination revealed chronic granulomatous inflammation with caseation necrosis. Ziehl-Neelsen staining demonstrated acid-fast bacilli. Periodic acid-Schiff staining was negative for fungal organisms. QuantiFERON-TB Gold assay was positive. Aspiration and culture of the adnexal lesion confirmed MTB.

Baseline laboratory investigations, including complete blood count, renal function, and liver function tests, were within normal limits.

The final diagnosis was ovarian tuberculosis with peritoneal involvement.

**Figure 2:**
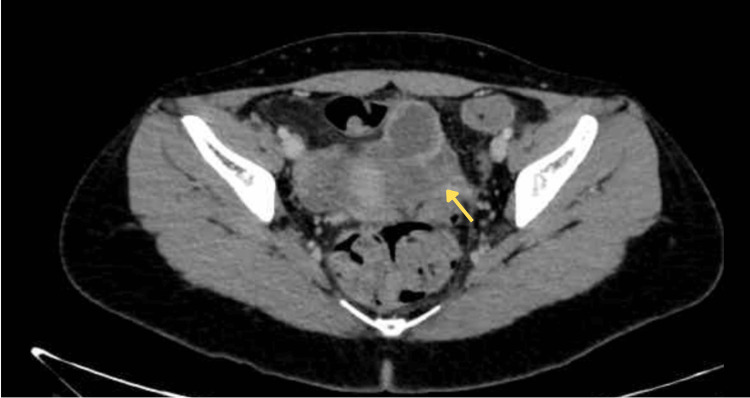
Follow-up contrast-enhanced axial CT scan obtained after initiation of anti-tuberculous therapy showing interval reduction in the size of the left adnexal lesion, consistent with therapeutic response.

The patient was started on first-line anti-tuberculous therapy consisting of isoniazid, RIF, ethambutol, and pyrazinamide. A CT scan performed after two months showed minimal radiological change (Figure 2); treatment was continued. A repeat serum CA-125 measurement obtained four months after treatment initiation showed a decrease from the initial value to 313.6 U/mL, suggesting a partial biochemical response consistent with reduction of inflammatory activity. Subsequent imaging demonstrated a reduction in lesion size. Clinically, the patient experienced resolution of abdominal pain and stabilization of weight. No significant adverse drug reactions were reported.

## Discussion

Ovarian tuberculosis represents a rare but clinically significant manifestation of FGTB. Ovarian involvement accounts for approximately 10-30% of cases of FGTB, although isolated ovarian disease is uncommon [1].

The pathogenesis of ovarian tuberculosis may occur through direct spread from the fallopian tubes, hematogenous dissemination, lymphatic spread, or reactivation of dormant bacilli [4]. In most reported cases, ovarian involvement occurs secondary to tubal or endometrial infection [6]. In our patient, no evidence of pulmonary tuberculosis was identified, suggesting either hematogenous dissemination or isolated genital involvement.

The clinical presentation of FGTB is often insidious and nonspecific. Patients may present with infertility, chronic pelvic pain, menstrual irregularities, or adnexal masses [1]. In this case, the patient presented with abdominal distension, severe hypogastric pain, and weight loss without respiratory symptoms.

Elevated CA-125 levels are commonly associated with epithelial ovarian carcinoma; however, they are not specific and may also occur in inflammatory or infectious conditions such as tuberculosis [7]. Although CA-125 levels in ovarian tuberculosis are usually reported below 500 U/mL [4], our patient exhibited a markedly elevated value of 637.4 U/mL, likely reflecting extensive peritoneal inflammation. This finding highlights the importance of tissue diagnosis before initiating oncologic therapy.

Imaging findings in ovarian tuberculosis are nonspecific. CT and magnetic resonance imaging may demonstrate complex adnexal masses, ascites, peritoneal thickening, and lymphadenopathy features that can closely mimic advanced ovarian carcinoma [8,9]. Previous case reports have described similar presentations in which ovarian tuberculosis was initially suspected to be ovarian malignancy due to the presence of adnexal masses, ascites, and elevated CA-125 levels [5].

Microbiological confirmation in FGTB remains challenging because of its paucibacillary nature. Acid-fast bacilli smear sensitivity ranges from 1% to 22%, while culture sensitivity ranges from 7% to 42% and may require several weeks for growth. Molecular assays such as Xpert MTB/RIF demonstrate high specificity but variable sensitivity [1]. Therefore, diagnosis frequently relies on a combination of clinical, radiologic, histopathologic, and microbiological findings.

Histopathological identification of granulomatous inflammation with caseous necrosis remains a key diagnostic feature, although other granulomatous conditions must be considered in the differential diagnosis [4]. In our case, histopathological findings combined with microbiological confirmation allowed definitive diagnosis and prevented unnecessary chemotherapy, and avoided potentially irreversible consequences such as extensive surgery, surgical menopause, and loss of fertility in a young patient.

Standard treatment for drug-sensitive extrapulmonary tuberculosis consists of a six-month regimen including an intensive phase with isoniazid, RIF, ethambutol, and pyrazinamide followed by a continuation phase with isoniazid and RIF. Surgical intervention is generally reserved for complications or diagnostic uncertainty [10].

Our patient demonstrated clinical and radiological improvement after initiation of anti-tuberculous therapy, consistent with outcomes reported in the literature.

## Conclusions

Ovarian tuberculosis remains a rare but important differential diagnosis in women presenting with complex adnexal masses, particularly in regions with intermediate or high tuberculosis prevalence. Its clinical and radiologic resemblance to ovarian malignancy poses significant diagnostic challenges and may lead to unnecessary invasive interventions if not carefully evaluated. Histopathological examination continues to play a crucial role in establishing the diagnosis in paucibacillary disease.

Early recognition and timely initiation of anti-tuberculous therapy are essential to prevent irreversible pelvic damage and long-term reproductive sequelae. This case underscores the importance of maintaining a high index of suspicion for genital tuberculosis when evaluating adnexal masses with atypical imaging features and elevated tumor markers in endemic settings. Ultimately, careful diagnostic evaluation can prevent misdiagnosis and ensure appropriate, life-changing treatment.
